# Eight Novel Microsatellite Loci in a Declining Grassland Songbird, the Bobolink (*Dolichonyx oryzivorus*)

**DOI:** 10.1002/ece3.71400

**Published:** 2025-05-07

**Authors:** Kathryn McGee, Noah G. Perlut, Steven E. Travis

**Affiliations:** ^1^ School of Biological Sciences University of New England Biddeford Maine USA; ^2^ School of Marine and Environmental Programs University of New England Biddeford Maine USA

**Keywords:** Hardy–Weinberg equilibrium, microsatellite, migratory songbird, non‐coding DNA

## Abstract

Microsatellite loci in non‐coding regions of nuclear DNA provide an important tool for genetic diversity research, particularly when comparing relatedness between individuals. While microsatellites have been developed for several species within the Blackbird family (*Icteridae*), no species‐specific microsatellite primers for genetic analysis of Bobolinks ( *Dolichonyx oryzivorus* ) exist. Like many grassland songbirds, Bobolink populations are in decline, and accessible genetic analysis tools are therefore critical for understanding population dynamics. This paper introduces ten new, polymorphic microsatellite loci, at least eight of which should provide a reliable tool for the future study and monitoring of Bobolink genetics. These hypervariable and thus highly informative loci were amplified in 152 adult individuals from a well‐studied population in Shelburne, Vermont, USA.

## Graphical abstract

This paper introduces ten new polymorphic microsatellite loci for genetic analysis of Bobolinks ( *Dolichonyx oryzivorus* , Family Icteridae), which, like many grassland songbirds, are declining across their native range in North and South America. The informativeness of these hypervariable loci was confirmed based on the genotyping of 152 adult individuals from a well‐studied population in Shelburne, Vermont, USA. These loci will provide an important tool for assessing relatedness between individuals and monitoring population dynamics over time.

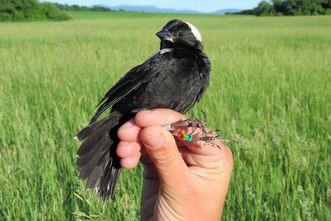

## 1 Introduction

**FIGURE 1:**
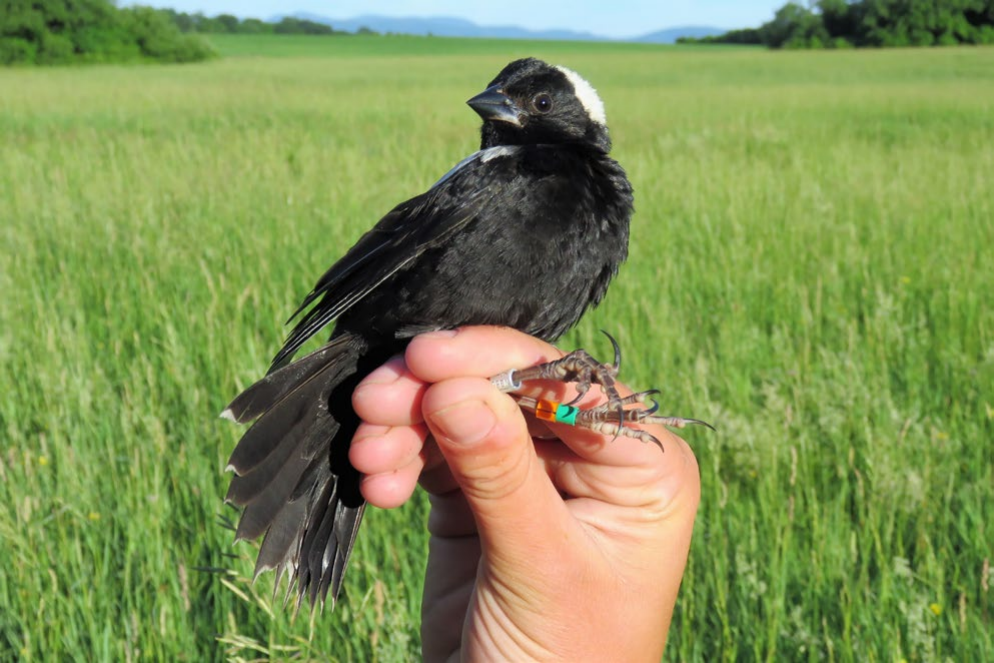
A male bobolink captured and banded on the study site in Shelburne, VT, USA.

Bobolinks ( *Dolichonyx oryzivorus* ; Figure 1) are long‐distance migratory songbirds, traveling over 6000 miles each year between their North American breeding grounds and South American wintering grounds (Renfrew et al. 2015, 2019). Bobolinks had an estimated global population of 10 million individuals in 2020 (Partners in Flight 2020), but the population is projected to decrease by 50% over the next 48 years (Partners in Flight 2021). As a grassland obligate species, habitat loss and mortality caused by agricultural management (Perlut et al. 2006; McGowan et al. 2021) present ongoing threats across their distribution (Renfrew et al. 2015, Sauer et al. 2022).

As the global population continues to decline, research to improve our understanding of Bobolink population genetics has investigated both extra‐pair paternity (White et al. 2021) and population structure (Renfrew et al. 2021). While researchers have found recent success in utilizing single‐nucleotide polymorphism (SNP) markers to characterize Bobolinks at the population level (Renfrew et al. 2021; Perlut et al. 2023), effective single‐sequence repeats (SSRs), also known as microsatellite markers, for Bobolinks, which are particularly important for gauging pairwise relatedness, are currently lacking. While both types of genetic tools have the potential to provide important genetic insights into Bobolink biology, microsatellite markers have an advantage over SNPs for reconstructing parental or individual relationships due to their increased polymorphism per locus and correspondingly higher rates of heterozygosity (Hauser et al. 2021).

White et al. (2021) analyzed Bobolink DNA with microsatellite loci from related species within the Icteridae family: Red‐winged Blackbird ( *Agelaius phoeniceus* ), Brown‐headed Cowbird ( *Molothrus ater* ) and Great‐tailed Grackle ( *Quiscalus mexicanus* ). They attempted to use seven loci, but three loci failed to adhere to Hardy–Weinberg equilibrium frequencies, indicating the possibility of null alleles; therefore, only four loci were included in their study (White et al. 2021). Given these challenges, we aimed to advance current and future population genetics study of the Bobolink by identifying species‐specific microsatellite loci.

Here we describe the development of a novel set of microsatellite markers for assessing genetic variation in the Bobolink. Microsatellites typically represent neutral markers in non‐coding regions of nuclear DNA, are traditionally used in genetic diversity studies as they are inherited in a standard Mendelian fashion and are generally outside the influence of natural selection (Kirk and Freeland 2011). By increasing the number of functional microsatellite loci for use in the Bobolink, we aim to improve our ability to investigate genetic relationships within this rapidly declining species.

## 2 Methods

### 2.1 Field Methods

We obtained blood samples from adult Bobolinks caught during the 2019 breeding season at Shelburne Farms (44.395168, −73.247001) in Shelburne, Chittenden County, Vermont, USA, as part of the ongoing Bobolink Odyssey project. Appropriate animal care was approved under the University of New England IACUC protocol number UNE010‐2009, and banding under U.S. federal permit 17610#23540. Since 2002, the Bobolink Odyssey has investigated the conservation and full life‐cycle of Bobolinks. Individuals were captured in 12‐m mist nets using song playback or by placing nets around active nests. After each adult bird was given a US Geological Survey band, 50 μL blood samples were extracted from the brachial vein and stored at −20°C on Whatman filter paper (grade P8—coarse). We extracted total genomic DNA using DNeasy Blood and Tissue Kits following the “Isolation of Total DNA from FTA and Guthrie Cards” protocol developed by the manufacturer (QIAGEN Inc., Germantown, Maryland). Of the 152 adults captured from the 2019 population, we collected blood samples from 133 individuals. For 19 adult birds missing blood samples, we utilized blood samples collected during previous seasons (2015–2018).

### 2.2 Primer Development

Microsatellite primer pairs were initially developed from eight randomly selected individual Bobolink samples with no known kinship based on nesting records. Microsatellite primer development following Wang et al. (2018) was performed by CD‐genomics (Shirley, NY) utilizing next‐generation, paired‐end sequencing on the Illumina platform (Illumina, San Diego, California, USA). This process generated an initial 5 Gigabytes of data assembled with the Stack package prior to searching for microsatellite loci using MISA software, where any locus with more than five repeats of 3–5 bp was considered a candidate. Tetranucleotide repeats were particularly sought for their higher mutation rates and correspondingly greater polymorphism. Primer pairs were developed from the flanking sequences of 60 loci using Primer3 software and then screened for their specificity and level of polymorphism by electrophoresing fluorescently labeled PCR products on an ABI 3730XI Genetic Analyzer. PCRs used standard melting and elongation temperatures/durations, along with annealing temperatures of 60°C applied for 1 min per cycle. Primers amplifying low quality, improperly sized, or non‐specific sequences or sequences that were lacking in polymorphism were discarded, leaving 10 highly polymorphic loci for further screening. All 10 sequences have been deposited in GenBank under accession number PRJNA1245395.

For the purposes of screening the entire adult Bobolink population from Shelburne, VT, forward primers received one of four fluorescent labels (Table 1). Touchdown polymerase chain reaction (PCR) protocols were performed in an Eppendorf Mastercycler (Hamburg, Germany) and used a total volume of 25 μL per amplification containing 1 μL (~50 ng) DNA, 0.5 units of Invitrogen Taq DNA Polymerase (Recombinant) in 1× PCR buffer (ThermoFisher Scientific, Waltham, Massachusetts), 4 mM MgCl_2_, 200 μM dNTPs, 1 μM of both forward and reverse primers, and 0.1 μg/μL BSA. Thermal cycler conditions used annealing temperatures optimized by the developer for each primer pair (Table 1) and included an initial denaturation at 96°C for 2 min; 5 cycles at 96°C for 20 s, primer annealing for 30 s, and 72°C for 1 min; 20 cycles at 96°C for 20 s, primer annealing for 30 s, and 72°C for 1 min; 10 cycles where the annealing temperature decreased by 0.5°C each cycle; and then 10 cycles at the touchdown annealing temperature (original annealing temp—10°C); followed by a final extension at 72°C for 1 min. PCR products were visualized for proper size and yield through electrophoresis using 2% agarose. Fragment analysis was performed at the Keck DNA Sequencing Facility at Yale University using an Applied Biosystems 3730× capillary DNA Analyzer. Chromatograms were scored manually for allele sizes using Genemapper V. 5 (Applied Biosystems, Foster City, CA, USA).

**TABLE 1:** Microsatellite loci developed for bobolink (*Dolichonyx oryzivorus*), including primer sequence and annealing temperature (*T*_*a*_).

SSR locus	*T*_*a*_	Repeat motif	Forward primer	Reverse primer	Reference length	Fluorescent label
BOBO‐01	60	(AAAC)_12_	TTTACACTTTCACTTTCCACCTCTG	AGATTTTGTGTTGTGTCCTGTGAGT	298	6‐FAM
BOBO‐02	59	(CATC)_13_	TTCAGACCTTCCATTCAGCAAA	GGGCAAGAGTGGGGTTCAA	230	VIC
BOBO‐03	57	(ATAG)_12_	CTCGGTAGTTTCCTCTGTTTGAT	CCCAGCCTGCCCTTAATAC	226	PET
BOBO‐04	55	(TAGT)_10_	ACCACCACCCTGAAAGCATA	CATTGGTTTTAATTTTGCAGCA	211	6‐FAM
BOBO‐05	57	(TGAA)_11_	TGGACTAATACACAAAGTAAAGCAAG	TGTCACCCCGTTTCAGCC	279	NED
BOBO‐06	58	(CAGT)_10_	CTGAGCTGCCATGCTCTTAAC	TTCCATAGGTCATTCTTTTCTCTCT	234	VIC
BOBO‐07	60	(ATTC)_11_	TTTCTTACAGGGTCTTACTCCAACA	ATCTTCCTTCAGTCCTTTCAGTCTC	275	NED
BOBO‐08	58	(TGAA)_13_	GACAGAAATGGTTTGGTTATGTCA	ATCCCTTCCTATGTGTTGAGCA	236	VIC
BOBO‐09	60	(AGGA)_10_	AGAGGATTTTACAGGGCAAGGA	AGAGCAGCATTAGGACTGGAGAT	228	NED
BOBO‐10	61	(TATC)_11_	GCCTGATCCTTATCCTCAGAGC	GCAGAGAAAGACATACCCACAGC	195	6‐FAM

## 3 Statistical Analysis and Results

To analyze the full potential of these new microsatellite loci for characterizing genetic variation in Bobolink populations, we first checked for evidence of null alleles, scoring errors, deviations from expected Hardy–Weinberg equilibrium (HWE) frequencies, and linkage disequilibrium among loci using the entire Vermont sample. For these assessments we first omitted any individuals with alleles well outside of the expected size range for each locus (i.e., > 50 bp beyond the most similarly sized allele). Using Micro‐Checker 2.2.3 (Van Oosterhout et al. 2004) we found one locus (BOBO‐07) with a predicted null allele frequency > 0.05 (0.0585). On the other hand, Micro‐Checker detected no evidence of large allele dropout, stutters, or scoring errors. Using GENEPOP 4.7.5 (Rousset 2008) only one locus, BOBO‐10, was found to deviate significantly from HWE (*p* = 0.000) using a Bonferroni adjusted alpha level of 0.005. In addition, we identified possible linkage disequilibrium between BOBO‐07 and BOBO‐06 (*p* = 0.0496) and between BOBO‐04 and BOBO‐09 (*p* = 0.0462), although these associations were non‐significant relative to a Bonferroni adjusted alpha level of 0.001.

We calculated standard genetic metrics with GenAlEx (Peakall and Smouse 2006, 2012; Table 2). All 10 loci were polymorphic, with 7 to 18 alleles identified for each locus. Observed and expected heterozygosity for each locus ranged from 0.664–0.847 ($\bar{x}$ = 0.7897) and from 0.696–0.886 ($\bar{x}$ = 0.7974), respectively. The average inbreeding coefficient, *F*_*IS*_, across all loci was not significantly different from zero at −0.0021, which, along with no deviation from Hardy‐Weinberg equilibrium, indicates an outbred population. This is lower than the previous inbreeding coefficient calculated for the Vermont population using SNPs (*F*_*IS*_ = 0.1442) (Renfrew et al. 2021). This difference in *F*_*IS*_ values may have been caused by sample size or temporal differences between our respective studies, with the SNP study utilizing individuals from the 2003–2004 and 2011 breeding seasons (*n* = 40) compared to our larger sample of adults from the 2019 breeding season (*n* = 152). In general, inbreeding estimates are rendered more accurate by the relative ease with which large numbers of SNP loci can be genotyped from across the genome compared to microsatellites (Hoffman et al. 2014), so it is not uncommon for estimates to differ between the two approaches (e.g., Pérez‐González et al. 2023).

**TABLE 2:** Summary of genetic diversity in the Shelburne, VT, Bobolink population (*N* = Number of genotypes successfully scored, *Ho* = Observed heterozygosity, *He* = Expected heterozygosity, and *F*_*IS*_ = Inbreeding coefficient).

SSR locus	*N*	Size range (bp)	Number of alleles observed	Effective number of alleles	*Ho*	*He*	*F*_*IS*_
BOBO‐01	152	256–280	7	3.289	0.664	0.696	0.045
BOBO‐02	147	190–234	9	4.595	0.803	0.782	−0.026
BOBO‐03	152	192–244	11	4.550	0.743	0.780	0.047
BOBO‐04	138	165–205	13	4.209	0.819	0.762	−0.074
BOBO‐05	150	228–322	18	8.738	0.847	0.886	0.044
BOBO‐06	139	195–231	14	5.780	0.842	0.827	−0.018
BOBO‐07	143	243–287	11	5.143	0.720	0.806	0.106
BOBO‐08	147	194–230	10	6.361	0.844	0.843	−0.001
BOBO‐09	133	186–226	12	6.996	0.842	0.857	0.017
BOBO‐10	150	158–190	9	3.768	0.773	0.735	−0.053

## 4 Conclusions

Of the ten microsatellite loci developed for Bobolinks, eight loci (BOBO‐01, −02, −03, −04, −05, −06, −08, −09) are ultimately appropriate for future analysis of the Shelburne, Vermont, breeding population, with the potential for use on populations spanning the entire species' distribution. We recommend loci BOBO‐07 and BOBO‐10 be approached with caution in future work as they displayed evidence of null alleles and a deviation from HWE frequencies, respectively. The high levels of observed heterozygosity among all loci suggest these new primers will be highly informative tools for genetic analysis, particularly relatedness studies (Hauser et al. 2021). In the future, these polymorphic loci will be utilized to further investigate population dynamics in the species, as well as heritability of physical and behavioral traits by correlating molecular marker and trait similarity (e.g., Ritland 1996). In an effort to conserve this declining species, expanding our toolbox of genetic techniques will allow insights to further inform ongoing management and conservation efforts.

## Author Contributions

**Kathryn McGee:** conceptualization (supporting), data curation (lead), formal analysis (lead), funding acquisition (lead), methodology (supporting), writing – original draft (lead). **Noah G. Perlut:** conceptualization (lead), project administration (lead), supervision (lead), writing – review and editing (supporting). **Steven E. Travis:** conceptualization (supporting), formal analysis (supporting), methodology (lead), project administration (supporting), resources (supporting), supervision (supporting), writing – review and editing (lead).

## Conflicts of Interest

The authors declare no conflicts of interest.

## Data Availability

The data that support the findings of this study are openly available in Open Science Framework at http://doi.org/10.17605/OSF.IO/WP5JB.
